# Secular trends in risk factors for adolescent anxiety and depression symptoms: the Young-HUNT studies 1995–2019, Norway

**DOI:** 10.1007/s00787-024-02373-2

**Published:** 2024-04-05

**Authors:** Morten Austheim Krokstad, Erik Sund, Vegar Rangul, Adrian Bauman, Craig Olsson, Ottar Bjerkeset

**Affiliations:** 1 Faculty of Health Sciences and Nursing, Nord Universitet-Levanger Campus, Levanger, Norway; 2 Department of Mental Health, Norwegian University of Science and Technology, Trondheim, Norway; 3 Department of Public Health and Nursing, HUNT Research Centre, Norwegian University of Science and Technology, Levanger, Norway; 4 Levanger Hospital, Nord-Trøndelag Hospital Trust, Levanger, Norway; 5 Murdoch Children’s Research Institute, Centre for Adolescent Health, The Royal Children’s Hospital Melbourne Victoria, Parkville, Australia; 6 School of Psychology, Faculty of Health, Centre for Social and Early Emotional Development, Deakin University, Burwood, VIC Australia; 7 Department of Paediatrics, University of Melbourne, Parkville, VIC Australia; 8 Prevention Research Collaboration, Sydney School of Public Health, The University of Sydney, Sydney, Australia

**Keywords:** Anxiety and depression symptoms, Risk factors, Adolescence, Decennial trends, Young-HUNT, Epidemiology

## Abstract

In recent decades, increases in mental health problems in adolescents have been reported from several large population-based surveys. This raises questions about changes in underlying risk and protective factors that can inform future intervention strategies. Population data were collected from 1995 to 2019 in three waves of the Young-HUNT studies in Norway to map decennial trends in the prevalence of established risk factors for, and their associations with, adolescent mental health problems. All adolescents (aged 13–19 years) attending lower and upper secondary school in the county of Trøndelag were invited, representing three historical cohorts of 25,245 unique adolescents. Mental health problems (HSCL-5) and established mental health risk factors were self-reported. Using a generalized linear model and linear regression, we calculated changes in relative and absolute differences between risk factors and mental health problems. Overall, the prevalence of established risk factors for mental health problems in adolescence increased markedly between 1995 and 2019, especially in girls. Prominent increases were observed for fatigue, bullying, musculoskeletal pain and migraine, loneliness, and overweight. Furthermore, with the exception of excess alcohol use and family economy, associations between each risk factor and adolescent mental health problems strengthened over the same time span in girls, but less among boys. Our findings suggest that several modifiable risk factors for poor mental health in adolescence are increasing, especially among girls, and should be targeted in community, school, and in clinical settings.

**Supplementary Information:**

The online version contains supplementary material available at 10.1007/s00787-024-02373-2.

## Introduction

There has been a striking increase in self-reported symptoms of anxiety and depression in Norwegian adolescents over the last 2 decades [1]. Similar trends have been reported elsewhere, supported by European [2], Asian [3], American [4, 5], and Australian findings [6]. This represents a global public health concern [7] as mental health problems are associated with numerous adverse psychosocial and individual outcomes throughout life, including education and unemployment [8], psychosomatic health complaints, and a lowered threshold for relapse of anxiety disorders and major depression [9].

Prevalence estimates of both mental health problems and disorders vary considerably (0.2–24% for depressive and anxiety disorders and 9–22% for psychological distress) across studies and study settings [7, 10–13]. Worldwide, it has been estimated that 10% to 20% of adolescents live with a diagnosed mental health condition [14], and that they account for approximately 13% of the global burden of disease and injury in 10–19 years old [15]. Mental health problems arise from interaction between risk and protective factors [16], some of which are (relatively) constant, yet others may be modifiable, and may vary greatly through the teenage years. Literature indicates that bullying and loneliness represent strong and established predictors for mental health problems [17–19], while following recommendations regarding healthy sleep, increased physical activity and reduced sedentary behaviours have shown strong and favourable associations with mental health benefits in adolescence. Apart from age, gender, SES, and, to some extent, adverse life events (ALEs), most known determinants/risk factors are considered modifiable in either societal, familial, or individual environments. However, less is known regarding the time trends in the risk factors for mental health problems in adolescence during recent decades.

The rapid rise in self-reported mental health problems, self-harm, and suicidal behaviours in young people implies possible changes in the underlying risk and protective factors. Typically, mental health problems emerge and often peak in adolescence and early adulthood [20], making adolescence a critical opportunity for early preventive interventions through educational system and through early treatment in primary and specialized care.

The aim of this study is to present decennial trends in risk factors potentially driving the increasing levels of anxiety and depression symptoms in Norwegian adolescents. We use a serial entry design, measuring individual, relational, and contextual data in three different population-based adolescent cohorts from 1995 to 2019 to examine (1) change in the prevalence of risk factors for mental health over time, and (2) changes in the relative and absolute differences between these risk factors and symptoms of anxiety and depression in girls and boys between 1995 and 2019.

## Methods

### Data and study population

Data were obtained from more than 25,000 participants aged 13–19 years in three waves of the Young-HUNT (YH) study, Norway: YH1 in 1995–97 (*n* = 9140, 89.6% response rate); YH3 in 2006–08 (*n* = 8203, 78.4% response rate); and YH4 in 2017–19 (*n* = 8124, 75% response rate). The surveys included self-reported questionnaires covering a wide range of aspects of physical and mental health, quality of life, lifestyle, and behavioural factors. All detailed instruments, variables, and response options are on the webpage for the HUNT Research Centre (https://www.ntnu.edu/hunt).

### Measurements

#### Dependent variable (anxiety and depression symptoms)

Psychological distress was assessed using a Norwegian validated and reliable short version of the Hopkins Symptom Checklist (HSCL-5), in all three waves of YH [21]. HSCL-5 is a feasible and succinct screening tool for anxiety and depression symptoms in large populations. Short versions of HSCL have shown good psychometric properties across adolescent and adult populations [22, 23] compared to longer versions, and have therefore been widely used in Norwegian population studies [24–26]. Questions about anxiety and depression symptoms in the previous 2 weeks were asked (feeling tense, anxious, hopeless, sad, and worried). Responses were scored from ‘not at all bothered’ (1 point) to ‘extremely bothered’ (4 points) on a Likert-scale, with item mean scores based on all five questions. Missing data ranged from 2.6 to 6.7% in the YH waves.

#### Independent variables (risk factors for anxiety and depression)

##### Individual factors

*Self-perceived health* was assessed by a single item in all three waves: the responses were dichotomized into: “Poor” and “Good” [27]. Missing data ranged from 0.9 to 1.7% across all waves.

*Fatigue* was assessed by one item from The Subjective Well-Being Scale in YH1 and 3 [28], and one item from the Chalder Fatigue Scale (CFS) [29] in YH4. We dichotomized the response options into “Strong/fit” and “Tired/worn out”. Missing data ranged from 1.0 to 2.8%.

*Excess alcohol use* was measured with a single item originating from Health Behaviour in School-aged Children (HBSC) [30]. We dichotomized the response options into “Excessive alcohol intoxication” (drunk > 10 times) and “ < 10 times” [31]. Missing ranged from 2.6 to 5.3%.

*Chronic pain* was assessed by asking two questions about how often participants have experienced musculoskeletal pain and whether they had migraine diagnosed by a doctor. Both questions were merged and responses were dichotomized into “Frequently/often” and “Seldom/never [32]. Missing ranged from 0.6 to 1.4%.

*Physical activity* level was assessed by a validated questionnaire in which participants reported their weekly frequency of physical activity [33]. The responses were dichotomized into “physically inactive” and “physically active”, based on international (WHO) recommendations [34]. Missing ranged from 1.2 to 1.8%.

*Sleep problems evening* was assessed by one question in all three YH waves. The responses were dichotomized into “Never/seldom” and “Sometimes/frequently” [35]. Missing ranged from 1.3 to 5.3%.

*BMI* was measured using a standard definition for adolescent overweight and obesity, adjusted for age and sex (ISO-BMI). “*Overweigh*t” and “*Obese*” categories were defined using the International Obesity Task Force cut-off values (International Organization for Standardization BMI) for children and adolescents [36].

##### Relational factors

*Being bullied* was assessed by one question in YH1 and 3, and one near identical item from the Strength and Difficulties questionnaire [37] in YH4. The responses were dichotomized into “Yes” and “No” [38]. Missing ranged from 1.9 to 10.0%.

*Loneliness* was assessed by the same question in all three YH studies. The responses were dichotomized into “Often/very often” and “Never/rarely/sometimes” [39]. Missing ranged from 1.1 to 8.4%.

*Family cohesion* was assessed by 3 items from the Resilience Scale for Adolescents (READ) [40], available in both YH3 and 4. READ has been validated across different cultural samples and settings, and demonstrates sound psychometric properties [41]. Responses were merged and dichotomized into “High family cohesion” and “Low family cohesion”. Missing ranged from 4.5 to 6.9%.

*General practitioner (GP) visits last 12 months* were assessed by a single question in all three YH waves, and dichotomized into “ ≥ 1 time” and “ < 1”. Missing ranged from 3.3 to 10.7%.

##### Contextual factors

*Sex and age* were obtained from the Norwegian National Registry and further included in the HUNT studies. *Family financial situation* was measured by one question in YH3 and 4. The responses were dichotomized into “Worse” and “Same or better”, compared to other families (27). Missing ranged from 1.2 to 8.5%.

*Traumatic events/adverse life events* (ALE’s) were measured by 9 adapted items from the UCLA PTSD Reaction Index [42] available in YH3 and 4. The response options were merged and the total score were dichotomized into “Yes, one time or more” and “Never”. Missing ranged from 5.6 to 8.4%.

A detailed variable overview and missing values are found in supplementary Table 4.

### Statistical analysis

We calculated descriptive statistics as frequencies, percentages, and averages. We specified two types of regression models to study associations between the exposures and HSCL-5.

First, linear regression models (OLS) were used to estimate absolute differences (beta coefficients, B) in (standardized) mean HSCL-5 between exposure categories, and we report 95% confidence intervals based on 2000 bootstrapped samples. Second, generalized linear models (GLMs) with a gamma distribution and a log-link function were used to estimate differences in (log of) means in HSCL-5 between exposure categories. Coefficients were exponentiated and is interpreted as a relative measure of difference (relative risk, RR). Estimates are reported with 95 percent confidence intervals (95% CI). The RR represents the ratio between means, whereas the beta coefficients represent the difference between standardized mean HSCL across exposure categories. To test for linear trends across survey waves, interaction terms between exposures and survey waves were specified. Analyses were conducted for each sex separately and we adjusted for continuous age centred at age 16 in each wave. Exposures that showed statistically significant changes (1995–2019) for both absolute and relative differences were included in the Discussion. All data management and analyses were conducted using Stata version 17 (Statacorp, 2021).

## Results

Table 1 presents descriptive characteristics, changes in mean HSCL-5 scores (symptoms of anxiety and depression) and prevalence of established risk factors for mental health problems in three historical adolescent cohorts (13–19 years) 1995–2019. Mean HSCL-5 scores increased from 1.35 to 1.44 in boys and from 1.65 to 1.95 in girls from YH 1 to YH4.

**Table 1:** Descriptive characteristics of three historical adolescent cohorts; Young-HUNT1 (1995–97), Young-HUNT3 (2006–08), and Young-HUNT4 (2017–19). Boys and girls aged 13–19 years

	YH1 (1995–97), *n* = 8980 (50.3% boys)		YH3 (2006–08), *n* = 8199 (49.1% boys)		YH4 (2017–19), *n* = 8066 (49.6% boys)	
	Boys *n* (%)	Girls *n* (%)	Boys *n* (%)	Girls *n* (%)	Boys *n* %)	Girls *n* (%)
Outcome—psychological distress						
Mean SCL-5 score (1 = not at all, 4 = very bothered)	1.34	1.56	1.35	1.65	1.44	1.95
Exposures—individual factors						
Mean age	16.0	16.1	15.9	15.8	16.1	16.0
Poor self-perceived health	437 (9.8)	531 (12.1)	375 (9.3)	499 (12.3)	445 (11.4)	672 (16.5)
Feeling fatigued (moderate/high degree)	1620 (36.3)	2234 (50.6)	1751 (44.3)	2454 (60.8)	1601 (41.0)	2631 (64.8)
Excess alcohol use (intoxicated > 10 times)	1391 (30.8)	1212 (27.2)	923 (22.7)	930 (22.5)	753 (19.0)	701 (17.1)
Musculoskeletal pain/migraine (≥ once a month)	854 (19.1)	1429 (32.2)	1269 (31.5)	1986 (48.4)	1602 (41.1)	2375 (58.5)
Physical inactivity (exercise 0–3 days a week)	3013 (67.7)	3427 (77.7)	2193 (54.7)	2607 (64.2)	2295 (59.0)	2731 (67.6)
Sleep problems (sometimes/often)	2008 (45.3)	2523 (57.3)	1961 (50.9)	2669 (66.8)	1709 (45.2)	2477 (62.1)
Overweight/obese (ISO-BMI)	992 (22.0)	907 (20.3)	1250 (30.7)	1186 (28.7)	1352 (34.1)	1174 (28.6)
Exposures—relational factors						
Bullied by peers (often/a lot)	97 (2.2)	83 (1.9)	205 (5.6)	120 (3.1)	307 (8.4)	258 (6.7)
Feeling lonely (often/very often)	187 (4.2)	346 (7.8)	251 (6.6)	484 (12.3)	250 (6.6)	569 (14.3)
Low family cohesion	–	–	1389 (36.6)	1702 (43.3)	1411 (37.8)	1594 (40.6)
Visited general practitioner (GP) last year (≥ 1 time)	1880 (45.2)	2417 (57.9)	1481 (40.8)	2105 (54.7)	2338 (62.2)	2931 (73.9)
Exposures—contextual factors						
Low perceived family economy (SES)	–	–	309 (8.3)	399 (10.2)	254 (6.5)	375 (9.2)
Experiencing adverse life events (ALEs) (≥ 1 times)	–	–	2857 (76.6)	3158 (81.5)	3097 (83.6)	3346 (86.3)

### Trends in established risk factors for mental health problems 1995–2019

Overall, we found an increasing trend of several well-known risk factors from YH1 to YH4. For girls, the most prominent were *fatigue* (50.6–64.8%), *bullying* (1.9–6.7%), *loneliness (*7.8–14.3%), *musculoskeletal pain and migraine* (32.2–58.5%), *overweight/obesity* (20.3–28.6%), and *GP visits* (57.9% to 73.9%). In contrast, we observe a decrease in *physical inactivity* (77.7–67.6%) and *excess alcohol use* (27.2–17.1%).

For boys, the largest increases from YH1 to YH4 were found in *bullying* (2.2–8.4%), *musculoskeletal pain and migraine* (19.4–41.1%), *overweight/obesity* (22.0–34.1%), and *GP visits* (45.2–62.2%). As in girls, a decrease in *excess alcohol use* was also observed in boys (30.8–19.0%).

In contrast, self-report of *poor self-perceived health, perceived family economy*, *low family cohesion, Adverse Life Events,* and *sleep problems* remained relatively stable from YH1 to YH4, in both boys and girls.

### Decennial trends in absolute differences (beta coefficients) in risk factors for mental health problems

Table 2 shows absolute differences (beta coefficients) in HSCL-5 for individual, relational, and contextual risk factors adjusted for age. For girls, the absolute difference in standardized HSCL between those reporting poor self-rated health compared with those reporting good self-rated health was 0.58 in YH1 which increased to 1.11 in YH4. Differences according to self-reported fatigue (0.58–1.05), musculoskeletal pain/migraine (0.42–0.76), physical inactivity (0.05–0.38), sleep problems (0.49–0.79), overweight/obesity (0.01–0.09), bullying (0.70–1.03), loneliness (1.18–1.59), GP visits (0.10–0.36), low family cohesion (0.64–0.75), and Adverse Life Events (0.32–0.47) were also increased.

**Table 2:** Decennial trends in absolute difference (beta coefficients) in established risk factors for psychological distress among girls and boys (13–19 years) across three historical cohorts from 1995 to 2019

Girls	Young-HUNT1 (1995–97)		Young-HUNT3 (2006–08)		Young-HUNT4 (2017–19)		
	Beta coeff	95% CI	Beta coeff	95% CI	Beta coeff	95% CI	ptrend
Exposures—individual factors							
Poor self-perceived health (ref = good)	0.58	[0.48–0.69]	0.79	[0.67–0.90]	1.11	[0.99–1.22]	***
Feeling fatigued (ref = not feeling fatigued)	0.58	[0.53–0.63]	0.69	[0.64–0.75]	1.05	[0.97–1.12]	***
Excess alcohol use (ref = < 10 times)	0.26	[0.19–0.34]	0.15	[0.06–0.25]	0.33	[0.21–0.45]	0.08
Musculoskeletal pain/migraine (ref = seldom/never)	0.42	[0.36–0.48]	0.58	[0.52–0.64]	0.76	[0.68–0.83]	***
Physical inactivity (ref = > 4 days a week)	0.05	[− 0.01–0.12]	0.17	[0.11–0.24]	0.38	[0.30–0.46]	***
Sleep problems (ref = seldom/never)	0.49	[0.44–0.54]	0.64	[0.59–0.70]	0.79	[0.72–0.87]	***
Overweight/obese (ref = normal/underweight)	0.01	[− 0.06–0.08]	0.14	[0.07–0.22]	0.09	[− 0.00–0.18]	*
Exposures—relational factors							
Bullied by peers (ref = not bullied)	0.70	[0.43–0.96]	1.12	[0.87–1.37]	1.03	[0.86–1.20]	*
Feeling lonely (ref = not lonely)	1.18	[1.05–1.30]	1.40	[1.29–1.50]	1.59	[1.48–1.70]	***
Visiting GP last year (ref = not visited)	0.10	[0.04–0.15]	0.24	[0.18–0.31]	0.36	[0.27–0.45]	***
Low family cohesion (ref = good)			0.64	[0.58–0.70]	0.75	[0.67–0.83]	*
Exposures—contextual factors							
Experienced ALE’s (ref = not experienced)			0.32	[0.24–0.39]	0.47	[0.36–0.57]	**
Low family economy (ref = better/same as others)			0.67	[0.55–0.80]	0.81	[0.67–0.96]	0.11
Boys	Beta coeff	95% CI	Beta coeff	95% CI	Beta coeff	95% CI	ptrend
Exposures—individual factors							
Poor self-perceived health (ref = good)	0.49	[0.39–0.59]	0.56	[0.45–0.67]	0.54	[0.42–0.66]	0.39
Feeling fatigued (ref = not feeling fatigue)	0.47	[0.43–0.52]	0.39	[0.34–0.44]	0.64	[0.58–0.70]	**
Excess alcohol use (ref = < 10 times)	0.04	[− 0.02–0.10]	0.14	[0.06–0.22]	0.05	[− 0.05–0.15]	0.23
Musculoskeletal pain/migraine (ref = never/seldom)	0.37	[0.31–0.43]	0.38	[0.33–0.44]	0.48	[0.42–0.54]	**
Physical inactivity (ref = > 4 days a week)	0.05	[0.00–0.09]	0.10	[0.05–0.14]	0.14	[0.08–0.19]	*
Sleep problems (ref = seldom/never)	0.39	[0.34–0.43]	0.40	[0.36–0.45]	0.54	[0.49–0.60]	***
Overweight/obese (ref = normal/underweight)	0.01	[− 0.04–0.06]	0.11	[0.05–0.16]	0.00	[− 0.06–0.06]	0.45
Exposures—relational factors							
Bullied by peers (ref = not bullied)	0.84	[0.62–1.05]	0.60	[0.45–0.75]	0.41	[0.29–0.54]	**
Feeling lonely (ref = not lonely)	1.16	[1.00–1.33]	1.19	[1.05–1.34]	1.40	[1.24–1.57]	0.07
Visiting GP last year (ref = not visited)	0.12	[0.08–0.17]	0.19	[0.14–0.24]	0.27	[0.21–0.33]	***
Low family cohesion (ref = good)			0.37	[0.32–0.43]	0.49	[0.43–0.55]	**
Exposures—contextual factors							
Experienced ALE’s (ref = not experienced)			0.24	[0.19–0.28]	0.30	[0.23–0.37]	0.10
Low family economy (ref = better/same as others)			0.58	[0.46–0.69]	0.49	[0.33–0.64]	0.35

* =  < 0.05

** =  < 0.01

*** =  < 0.001

For boys, we found evidence for small changes in absolute differences for fatigue (0.47–0.64), musculoskeletal pain/migraine (0.37–0.48), physical inactivity (0.05–0.14), sleep problems (0.39–0.54), GP visits (0.12–0.27), and low family cohesion (0.37–0.49). In contrast to other risk factors, we observed decreasing differences according to bullying and mental health problems from YH1 to YH4 (0.84–0.41).

The absolute differences were higher in all waves for all risk factors in girls, except for overweight/obesity, bullying, and GP visits in YH1. Differences were generally higher for all risk factors in all YH waves for girls, except for bullying, GP visits, physical inactivity, and loneliness in YH1.

### Decennial trends in relative differences (relative risk, RR) in risk factors for mental health problems

Results for the relative differences (Relative Risk, RR) are displayed in supplementary Table 3. For girls, those reporting poor self-rated health had 1.23 times higher HSCL than those reporting good self-rated health in YH1. In YH4, the relative risk increased to 1.36. Similarly, we observed increased relative differences in HSCL from YH1 to YH4 in fatigue (1.25–1.41), musculoskeletal pain (1.17–1.27), physical inactivity (1.02–1.13), sleep problems (1.21–1.29), and GP visits (1.04–1.12).

For boys, smaller increases from YH1 to YH4 was observed for fatigue (1.23–1.30), physical inactivity (1.02–1.13), sleep problems (1.19–1.25), and GP visit(s) (1.06–1.12) were observed. On the contrary, reported bullying declined (1.38–1.18).

Loneliness showed the highest relative risk in all YH waves for both sexes, but remained stable across waves for both girls (1.47–1.52) and boys (1.53–1.60).

## Discussion

The substantial increase in adolescent mental health problems in the Young-HUNT studies, particularly for girls, is consistent with global findings (2–7). Here, we demonstrate a trend over 2 decades in both the prevalence of key risk factors for poor mental health as well as the strength of association between these factors and mental health problems. After standardization of the HSCL-5, the most prevalent risk factors in 2019 were loneliness, poor self-perceived health, fatigue, and bullying, while they also showed the largest absolute increase from 1995–2019.

### Trends in established risk factors for mental health problems from 1995 to 2019

As proposed by Geoffrey Rose [43], even relatively small increases in the prevalence of risk factors can lead to substantial effects on the population risk factor distributions, particularly distributional changes that increase the prevalence of high-risk groups—making them of population health importance.

### Individual factors

In keeping with other international studies [44, 45], we observe similar prevalences and gender differences in *poor self-perceived health* (10–20%) Further, our data indicate that, among girls, self-rated health is also more strongly associated with psychological distress in YH4 compared to YH1.

*Fatigue* and tiredness are commonly reported in the U.S and Europe [46, 47], and affect between 7 and 45% of the adolescent population [48]. Our results along with an Australian study [49] indicate an even higher prevalence of fatigue. The increase in fatigue, and the strength of association between fatigue and mental health problems, is more prominent in girls than boys, consistent with other studies [48, 50].

*Musculoskeletal pain (MSP)/migraine* are relatively common in adolescence and particularly in girls [51, 52]. We observe similar increases in MSP in both genders, yet a stronger association with mental health problem in girls compared to boys from 1995 to 2019.

*Problems initiating sleep* affect about one in four adolescents in Europe and Canada [53], and are more problematic among girls than boys [53, 54]. Further, in contrast to many European and Scandinavian countries [55, 56], our findings show a relatively stable prevalence of sleep problems over 2 decades in both sexes. Nevertheless, associations between sleep and mental health problems increased over time in our study.

### Relational factors

In contrast to several European countries [57], our study indicates an increase in *bullying* in both sexes. However, bullying was relatively infrequent overall, and more prevalent in boys than girls [57, 58]. However, being bullied was increasingly related to symptoms of anxiety and depression from 1997 to 2019 in girls, while these associations decreased in boys.

*Feeling of loneliness* is now considered a public health problem [59], and prevalences range between 9 and 14% worldwide [60]. Some Scandinavian studies also suggest an increase in adolescent loneliness the last decades [61, 62], particularly in girls [63, 64], consistent with our findings. Further, we observe an increasing association between loneliness and mental health problems over time in both boys and girls.

Parallel to the increases in psychological distress, more adolescents had *visited their GP* from YH1 to YH4 in our study. Similar estimates have been reported in Australian and Irish adolescents [65, 66], and underlines worsening self-perceived health [67]. Although variations are expected due to differences in health care systems, a Canadian cross-sectional study found similar prevalence rates, but a decrease over time in GP visits and an increase over time for MHP (mental health professionals) visits [68]. Finally, according to our data, GP visits show stronger associations with anxiety and depression symptoms over time.

In sum, prominent risk factors are associated with trends in adolescent mental health, particularly in girls, and represent a cluster of modifiable individual, relational, and contextual health characteristics and behaviours.

### Methodological considerations: strengths and limitations

Several methodological issues in our study are worth considering [69]. When it comes to selection bias, all three HUNT studies used similar designs and sampling, and were collected 10–12 years apart, and showed high participation rates, which improve the generalisability of these findings. Although the HUNT catchment area is confined to small towns and rural areas, it is fairly representative of Norway [70].

Measurement bias is also worth mentioning when investigating cross-sectional studies over time. The HSCL-5 (outcome) has been used in all three HUNT studies. However, over 20 years, societal and cultural changes may have influenced awareness surrounding mental health, and, in turn, responses to mental health questions. These changes influence willingness to report and therefore could lead to inflated estimates over time. This may also apply to adolescents’ responses to exposure variables. Missing values were low (< 5%) for near all variables in all three YH waves, with higher missing rates among boys (e.g., 6.9% for boys compared to 4.8% for girls on HSCL-5 in YH4). Higher missing values (5–10%) were observed for more sensitive variables in both genders (e.g., bullying and trauma). While many of the questions/variables originate from well-known instruments and scales, such as Health Behaviour in School-aged Children (HBSC), Interpersonal violence, brief lifetime trauma screen, and HSCL-5, this study also used some less well-validated questions. Although the latter were repeated across YH1-4, they often make direct comparison to other studies difficult. Further, the YH questionnaires were developed and adapted over 3 decades; therefore, there are some minor differences in some of the scales and response options on the exposure variables/covariates used from 1995 to 2019. This was considered carefully during the operationalization and categorization of the variables. Unfortunately, we did not have comparable data/instruments from all three YH studies for school-level variables, social media/online gaming, and behaviour/externalizing problems—factors of great interest and linked to mental health problems in adolescents.

To the best of our knowledge, this is one of the largest studies to study decennial trends in correlates and risk factors for adolescent mental health problems. Finally, in an exploratory study of many possible risk factors for anxiety and depression, it is not feasible to thoroughly describe trends for each factor. We argue that well-designed qualitative studies of changes in mental health stigma and awareness, and semantic issues related to mental health, are needed to further understand these phenomena.

## Conclusions

Our findings suggest that adolescent self-reported health, loneliness, fatigue, bullying victimization, sleep problems, musculoskeletal pain, and GP visits are increasing and represent increasing and modifiable risk factors for poor mental health in adolescents. Efforts to prevent mental health problems will most likely require different intervention components, including families, schools, neighborhoods, voluntary organizations, as well as primary and specialized care settings.

## Supplementary Information

Below is the link to the electronic supplementary material.

Supplementary file1 (DOCX 24 KB)

Supplementary file2 (XLSX 19 KB)

## Data Availability

The variables generated and analysed during the current study are available upon request. Requests for access to the datasets should be directed to Morten Krokstad. E-mail: Morten.Krokstad@nord.no.
