# Supplementary material for: Secular trends in risk factors for adolescent anxiety and depression symptoms: the Young-HUNT studies 1995–2019, Norway
Source: Eur Child Adolesc Psychiatry. 2024 Apr 5;33(11):3819–27. doi: 10.1007/s00787-024-02373-2 (PMC11588762; doi:10.1007/s00787-024-02373-2)
Supplement: Supplementary file 1 — Supplementary file1 (DOCX 24 KB) [file 787_2024_2373_MOESM1_ESM.docx]

| **Table 3. Decennial trends in relative risk (RR) in established risk factors for psychological distress among girls and boys (13-19 years) across three historical cohorts from 1995-2019** | | | | | | | |
| --- | --- | --- | --- | --- | --- | --- | --- |
|  | **YH1 (1995-97)** | | **YH3 (2006-08)** | | **YH4 (2017-19)** | |  |
| **Girls** | **RR** | 95% CI | **RR** | 95% CI | **RR** | 95% CI | **ptrend** |
| **Exposures - individual factors** |  |  |  |  |  |  |  |
| Poor self-perceived health (ref= good) | 1,23 | [1,19-1,26] | 1,29 | [1,25-1,34] | 1,36 | [1,31-1,40] | ****** |
| Feeling fatigued (ref= not feeling fatigued) | 1,25 | [1,23-1,28] | 1,30 | [1,27-1,33] | 1,41 | [1,38-1,45] | ****** |
| Excess alcohol use (ref= < 10 times) | 1,09 | [1,06-1,12] | 1,10 | [1,02-1,09] | 1,10 | [1,06-1,14] | ***** |
| Musculoskeletal pain/migraine (ref= seldom/never) | 1,17 | [1,14-1,19] | 1,24 | [1,21-1,26] | 1,27 | [1,24-1,30] | ****** |
| Physical inactivity (ref= > 4 days a week) | 1,02 | [1,00-1,05] | 1,07 | [1,04-1,09] | 1,13 | [1,10-1,16] | ****** |
| Sleep problems (ref= seldom/never) | 1,21 | [1,19-1,23] | 1,28 | [1,25-1,31] | 1,29 | [1,26-1,32] | ****** |
| Overweight/obese (ref= normal/underweight) | 1,05 | [1,04-1,05] | 1,06 | [1,03-1,08] | 1,03 | [1,00-1,06] | .06 |
| **Exposures - relational factors** |  |  |  |  |  |  |  |
| Bullied by peers (ref= not bullied) | 1,27 | [1,18-1,37] | 1,42 | [1,33-1,51] | 1,34 | [1,27-1,40] | .15 |
| Feeling lonely (ref= not lonely) | 1,47 | [1,42-1,52] | 1,54 | [1,49-1,58] | 1,52 | [1,48-1,57] | ***** |
| Visiting GP last year (ref= not visited) | 1,04 | [1,02-1,06] | 1,10 | [1,07-1,12] | 1,12 | [1,09-1,15] | .96 |
| Low family cohesion (ref= good) | - |  | 1,26 | [1,23-1,28] | 1,25 | [1,22-1,28] | ****** |
| **Exposures - contextual factors** |  |  |  |  |  |  |  |
| Experienced ALE's (ref= not experienced) | - | - | 1,13 | [1,10-1,16] | 1,17 | [1,13-1,21] | .08 |
| Low family economy (ref= better/same as others) | - | - | 1,25 | [1,21-1,30] | 1,26 | [1,20-1,31] | .82 |
| **Boys (13-19 years)** | **RR** | 95% CI | **RR** | 95% CI | **RR** | 95% CI | **ptrend** |
| **Exposures - individual factors** |  |  |  |  |  |  |  |
| Poor self-perceived health (ref= good) | 1,22 | [1,19-1,26] | 1,26 | [1,21-1,30] | 1,23 | [1,18-1,28] | .65 |
| Feeling fatigued (ref= not feeling fatigued) | 1,23 | [1,21-1,25] | 1,19 | [1,16-1,21] | 1,30 | [1,27-1,33] | ****** |
| Excess alcohol use (ref= < 10 times) | 1,02 | [0,99-1,04] | 1,06 | [1,02-1,09] | 1,01 | [0,97-1,06] | .29 |
| Musculoskeletal pain/migraine (ref= seldom/never) | 1,17 | [1,14-1,20] | 1,18 | [1,16-1,21] | 1,22 | [1,19-1,25] | ****** |
| Physical inactivity (ref= > 4 days a week) | 1,02 | [1,00-1,04] | 1,04 | [1,02-1,06] | 1,06 | [1,03-1,09] | ***** |
| Sleep problems (ref= seldom/never) | 1,19 | [1,17-1,21] | 1,20 | [1,17-1,22] | 1,25 | [1,23-1,28] | ****** |
| Overweight/obese (ref= normal/underweight) | 1,01 | [0,99-1,04] | 1,05 | [1,03-1,08] | 1,00 | [0,97-1,03] | .74 |
| **Exposures - relational factors** |  |  |  |  |  |  |  |
| Bullied by peers (ref= not bullied) | 1,38 | [1,30-1,47] | 1,27 | [1,22-1,33] | 1,18 | [1,13-1,23] | ******* |
| Feeling lonely (ref= not lonely) | 1,53 | [1,47-1,60] | 1,55 | [1,49-1,61] | 1,60 | [1,53-1,68] | .13 |
| Visiting GP last year (ref= not visited) | 1,06 | [1,04-1,08] | 1,18 | [1,15-1,20] | 1,22 | [1,19-1,25] | ***** |
| Low family cohesion (ref= good) |  |  | 1,09 | [1,07-1,11] | 1,12 | [1,09-1,15] | .09 |
| **Exposures - contextual factors** |  |  |  |  |  |  |  |
| Experienced ALE's (ref= not experienced) | - | - | 1,12 | [1,09-1,14] | 1,14 | [1,10-1,18] | .28 |
| Low family economy (ref= better/same as others) | - | - | 1,26 | [1,21-1,31] | 1,20 | [1,15-1,26] | .13 |

|  |  |  |  |
| --- | --- | --- | --- |

sig. levels at: *** = <.001 ** = <.01 * = <.05
